# Reviewer acknowledgement 2013

**DOI:** 10.1186/1472-6750-14-9

**Published:** 2014-01-30

**Authors:** Dafne Solera

**Affiliations:** 1 BioMed Central, 236 Gray’s Inn, Road, London WC1X 8HB, UK

## Abstract

**Contributing reviewers:**

The editors of *BMC Biotechnology* would like to thank all our reviewers who have contributed to the journal in Volume 13 (2013).

## Reviewer acknowledgment

Mark Abbott

UK

Sabu Abdulhameed

India

Maja Adamska

Norway

Taby Ahsan

USA

Miguel Alcalde

Spain

Rosa Alduina

Italy

Jozef Anne

Belgium

Ben Appelmelk

Netherlands

Mehdi Arbabi Ghahroudi

Canada

Patrick Arbuthnot

South Africa

Yonathan Arfi

Israel

Hideyuki Arimitsu

Japan

Michaela Arndt

Germany

Ulrich Arnold

Germany

Ranjana Arya

India

Stéphane Aymerich

France

Ana Azevedo

Portugal

Christian Badr

USA

Marcelo Baeza

Chile

William Baldwin

USA

Zsófia Bánfalvi

Hungary

Zoltan Banki

Austria

Neil Barclay

UK

Jaouadi Bassem

Tunisia

Tarakdas Basu

India

Per Berglund

Sweden

Jean-Guy Berrin

France

Maurizio Bettiga

Sweden

Rama Bhadekar

India

Jasneet Bhullar

USA

Lars Mathias Blank

Germany

Barry Bochner

USA

Maria Jose Bonete

Spain

Jonas Borch

Denmark

Vilceu Bordignon

Canada

Nicole Borth

Austria

Dominique Böttcher

Germany

Loic Briand

France

Marcelo Brigido

Brazil

William Broughton

Germany

Frank Buchholz

Germany

Jochen Büchs

Germany

Bruno Buehler

Germany

Jan Buer

Germany

Kellie Burris

USA

Michael Butler

Canada

Robert Campbell

Canada

Nicolas Camper

UK

Lixiang Cao

China

Sofia Caretto

Italy

Melanie Carless

USA

Christopher Cazzonelli

Australia

Eda Celik

Turkey

Anju Chadha

India

Krishanu Chakrabarti

India

Yongchang Chang

USA

Guo-Qiang Chen

China

Jian Chen

China

Jun Chen

China

Yihua Chen

China

Alexander Chetverin

Russian Federation

Dennis Claessen

Netherlands

Udo Conrad

Germany

Greg Cook

New Zealand

Ana Coroadinha

Portugal

Pierre Cosson

Switzerland

Rosymar Coutinho de Lucas

Brazil

Sandor Cseh

Hungary

Rosa Cusido

Spain

Andre Damasio

Brazil

Robert Damoiseaux

USA

Guenther Daum

Austria

Robert Day

New Zealand

Teresa de la Rubia

Spain

Ario de Marco

Slovenia

Zeger Debyser

Belgium

Nicole Déglon

Switzerland

Frank DeLeo

USA

Vipin Deo

Japan

Jens Dernedde

Germany

Simon Christopher Deroles

New Zealand

Xianmin Diao

China

Lubbert Dijkhuizen

Netherlands

Mustafa Diken

Germany

Joseph Dillard

USA

Maria Margarida Diogo

Portugal

Tarek Dishisha

Sweden

Jinhua Dong

Japan

Francisco dos Santos

Portugal

Dion du Plessis

South Africa

Franck Duong

Canada

Charles Easley

USA

Abdou Elsharawy

Germany

Chijioke Emenike

Malaysia

Shymaa Enany

Japan

David Engelke

USA

Sabine Essler

Austria

Gert-Jan Euverink

Netherlands

Edgardo Farinas

USA

Jeanni Fehrsen

South Africa

Boris Fehse

Germany

Maria Fernandez Lobato

Spain

Jerome Ferrance

USA

Paul Fisher

USA

Judith Fliegmann

France

Diogo Fonseca-Pereira

Portugal

Annie Frelet-Barrand

France

Chunxiang Fu

USA

Guangsen Fu

USA

Douglas Fudge

Canada

Germaine Fuh

USA

Makoto Furutani-Seiki

UK

Melanie Galla

Germany

Sudeep George

USA

Rik Gijsbers

Belgium

Jordi Girones

Spain

Torsten Goldmann

Germany

Manuela Gomes

Portugal

Joao Goncalves

Portugal

Vijaya Gopal

India

Sailaja Gopalakrishnanchettiar Sivakamiammal

USA

Livia Goto Silva

Brazil

Mario Graos

Portugal

Dirk Grimm

Germany

Georg Guebitz

Austria

Shengjie Guo

China

Jacques Guyette

USA

John Hamill

Australia

Ulf Hanefeld

Netherlands

Donny Hanjaya-Putra

USA

Anders Johannes Hansen

Denmark

Israel Hanukoglu

Israel

Richard Harbottle

UK

Timm Harder

Germany

Mitsuru Haruki

Japan

Colin Harwood

UK

Mingyue He

UK

Jun Hiratake

Japan

Emilio Hirsch

Italy

Robert Anthony Hirst

UK

Mads Vilhelm Hollegaard

Denmark

Elizabeth Hood

USA

Dechamma Hosur Joyappa

India

Steven Howe

UK

Haiwei Huang

China

Shihshieh Huang

USA

Norikazu Ichihashi

Japan

Hiroyasu Inoue

Japan

Ander Izeta

Spain

Yves Jacob

USA

Jeanne Jacobs

New Zealand

Sharmili Jagtap

India

David Jakeman

Canada

Grzegorz Janusz

Poland

Laura Jarboe

USA

Jacob Jensen

USA

Antonio Jimenez

Spain

Shouguang Jin

USA

Robert Johnson

USA

Keisuke Kaji

UK

Vipin Kalia

India

Jeong-Hun Kang

Japan

Ajit Kanwal

India

Katy Kao

USA

Tatsuya Kato

Japan

Servé Kengen

Netherlands

Mariena Ketudat-Cairns

Thailand

Nawazi Khan

USA

Daisuke Kiga

Japan

Chunsung Kim

South Korea

Yeon-Gu Kim

South Korea

David Kirk

USA

Eric Klee

USA

Karl Klose

USA

Nobuhiko Kojima

Japan

Harald Kolmar

Germany

Anne-Brit Kolsto

Norway

Peter Kroth

Germany

Shuji Kubo

Japan

Mahesh Kulkarni

India

Hemant Kumar

India

Christos Kyratsous

USA

Nikolaos Labrou

Greece

Irina Lagutina

Italy

Ying Lai

USA

Uma Lakshmipathy

USA

Zhihong Lang

China

Oscar Lara-Velasco

USA

Nigel Larsen

New Zealand

Gerald LeBlanc

USA

Byong Lee

Canada

Choon-Taek Lee

South Korea

Hoyun Lee

Canada

Jaebeom Lee

South Korea

Jean Lee

USA

Sally Leys

Canada

Boon Lim

China

Jian-Zhong Liu

China

Wusheng Liu

USA

Matxalen Llosa

Spain

Lye Theng Lock

USA

Lili Lou

USA

Wen-Yong Lou

China

Fuping Lu

China

Astrid Rosa Mach-Aigner

Austria

Catarina Madeira

Portugal

Maheswaran Mani

India

David Mann

USA

Ian Marison

Ireland

Dirk Martens

Netherlands

Ludmila Martinkova

Czech Republic

Ligia Martins

Portugal

Hugh Mason

USA

Michael Maurer

Austria

Ana Mazotto

Brazil

Alexander Mellmann

Germany

Vishnu Menon

India

Florian Mertes

Germany

Jose Roberto Mineo

Brazil

Saroj Mishra

India

Csaba Miskey

Germany

Kentaro Miyazaki

Japan

Masaya Miyazaki

Japan

Dongyan Mu

USA

Ruiling Mu

USA

Jochen Mueller

Germany

Michael Müller

Germany

Ewen Mullins

Ireland

Maureen Murphy

USA

Serge Muyldermans

Belgium

Jill Myers

USA

Takeharu Nagai

Japan

Rekha Nair

USA

Akio Nakane

Japan

Hideo Nakano

Japan

Jahn Nesland

Norway

Donna Neumann

USA

Elke Nevoigt

Germany

Eric Nicholson

USA

Jing Ning

USA

Ken-Ichi Nishijima

Japan

Ahuva Nissim

UK

Elke Noens

Germany

Kenneth Noll

USA

Cory Nykiforuk

Canada

Carla Oliveira

Portugal

Maria José Oliveira

Portugal

Takeshi Omasa

Japan

Clarence Ongkudon

Malaysia

Linda Otten

Netherlands

Karin Overkamp

Netherlands

Sriram Padmanabhan

India

Christos Panagiotidis

Greece

Hongbo Pang

USA

Binod Parameswaran

India

Enoch Park

Japan

Luís Passarinha

Portugal

Jean Peccoud

USA

Maria Pedreno

Spain

Ren Peng

China

Yingjie Peng

USA

Suprasanna Penna

India

Bruno Pereira

Portugal

Laura Perin

USA

Clemens Peterbauer

Austria

Reuben Peters

USA

Sinisa Petrik

Czech Republic

Virginia Picanco

Brazil

Jorge Piedrahita

USA

Nuttaporn Pimpha

Thailand

Chris Porada

USA

Lisa Porter

Canada

Peter J Punt

Netherlands

Xu Qingsong

China

Chengxiang Qiu

USA

Jiayuan Quan

USA

João Queiroz

Portugal

Marko Radic

USA

KSMS Raghava Rao

India

Rama Rajaram

India

Sana Raouche

France

Eric Record

France

B Rajasekhar Reddy

India

Manfred Reetz

Germany

Qun Ren

Switzerland

Nick Roberts

New Zealand

Corinne Ronfort

France

Daniele Rosellini

Italy

Helene Rosenberg

USA

Ulrich Rothbauer

Germany

Marianne Rots

Netherlands

Scott Rudge

USA

Pasquale Saldarelli

Italy

Rangarajan Sampath

USA

Toshinori Sato

Japan

Christine Scaman

Canada

Axel Schambach

Germany

Joachim Schiemann

Germany

Stefan Schillberg

Germany

Thomas Schirrmann

Germany

Georg Schulz

Germany

Richard Scott

New Zealand

Bernhard Seiboth

Austria

Urartu Seker

Turkey

Susan Sharfstein

USA

Joseph Shiloach

USA

Carmen Simón-Mateo

Spain

Ankur Singh

USA

Sudhir Singh

India

Erik Snapp

USA

Hyuk Song

South Korea

Shiping Song

China

Irma Soria-Mercado

Mexico

Oliver Spadiut

Austria

Fabio Squina

Brazil

Juergen Stech

Germany

Uli Stingl

Saudi Arabia

Padmini Sudarshana

India

Weibo Sun

China

Petri Susi

Finland

Hidetoshi Tahara

Japan

Naping Tang

China

Nelson Tang

Hong Kong

Hiroshi Tarui

Japan

Keith Taylor

Canada

Ana Teixeira

Portugal

Carlos Termignoni

Brazil

Roger Thilmony

USA

Christina Thirlwell

UK

Robert Thomas

UK

Nuttha Thongchul

Thailand

Jim Thorsen

Norway

Hirekodathakallu Thulasiram

India

Kai Tittmann

Germany

Jiansong Tong

USA

Jorg Tost

France

Philippe Totte

France

William Trimble

Canada

Kouhei Tsumoto

Japan

Ossi Turunen

Finland

Christian Ukuru

Nigeria

Jitka Ulrichova

Czech Republic

Peter Uzoegwu

Nigeria

Marc van der Maarel

Netherlands

Geertje van Keulen

UK

Mirinda Van Kleef

South Africa

Jolanda van Munster

UK

Vinod Vathipadiekal

USA

Eirini Velliou

UK

Karuppannan Veluthambi

India

Robert Verpoorte

Netherlands

Stephan von Gunten

Switzerland

Constantinos Vorgias

Greece

Harald Wajant

Germany

Gerard Wall

Ireland

Chaozhan Wang

China

Huiqiang Wang

USA

Jiangxin Wang

China

Yun Wang

USA

Wilmore Webley

USA

Shiping Wei

China

David Wells

New Zealand

John Wheeler

USA

Nick Wierckx

Germany

Iain Wilson

Austria

Christoph Winkler

Singapore

Alexandre Wohlkonig

Belgium

Bernd Wollscheid

Switzerland

Dominic Wong

USA

Katarzyna Wrobel

Mexico

Mo Xian

China

Danke Xu

China

Wenxin Xu

USA

Montarop Yamabhai

Thailand

Soichiro Yamada

USA

Masayuki Yamato

Japan

Yi Yang

China

Cynthia Yiu

Hong Kong

Jianlan You

USA

Ronen Zaidel-Bar

Singapore

Barbara Zavan

Italy

Yitao Zeng

China

Nan Zhao

USA

Huanzhang Zhu

China

Limin Zhu

China

Maria Antonietta Zoroddu

Italy

